# Perception of the Adoption of Artificial Intelligence in Healthcare Practices Among Healthcare Professionals in a Tertiary Care Hospital: A Cross-Sectional Study

**DOI:** 10.7759/cureus.69910

**Published:** 2024-09-22

**Authors:** Adithyan N, Rupal Roy Chowdhury, Padmavathy L, Roshni Mary Peter, Anantharaman VV

**Affiliations:** 1 Community Medicine, SRM Medical College Hospital and Research Centre, SRM Institute of Science and Technology, Chengalpattu, IND

**Keywords:** adoption, artificial intelligence, attitude, barriers, healthcare professionals, knowledge, perception

## Abstract

Background: Artificial intelligence (AI) has the potential to transform healthcare practices, but the successful adoption of AI depends on the perception and acceptance of healthcare professionals. This study aimed to assess the understanding of AI concepts, recognize the attitude toward AI integration, and identify the barriers to AI adoption among healthcare professionals in a tertiary care hospital.

Methods: A cross-sectional study was conducted among 200 healthcare professionals, including doctors, nurses, and paramedics, in a tertiary care teaching hospital in Chengalpattu district, India. A semi-structured questionnaire was used to collect data on sociodemographic characteristics, knowledge, attitude, and perceived barriers to AI adoption. The chi-square test was used to analyze the associations between variables.

Results: The majority of the participants, i.e., 54% (n = 108), had moderate knowledge about AI adoption, while 48% (n = 96) had a low attitude toward it. Barriers to the adoption of AI in healthcare practices among healthcare professionals were low among the majority, i.e., 49% (n = 98), of the participants. A statistically significant association was found between knowledge and attitude (X² = 18.052, df = 4, p = 0.001), i.e., healthcare professionals with moderate knowledge levels had low attitudes toward the adoption of AI. A statistically significant association was also found between knowledge and perceived barriers (X² = 31.235, df = 4, p = 0.00), i.e., healthcare professionals with higher knowledge levels perceived lower barriers to the adoption of AI.

Conclusion: The study highlights the need for education and training to improve knowledge, foster positive attitudes, and address the perceived barriers to AI adoption among healthcare professionals. Future research should focus on developing targeted interventions to enhance the understanding and acceptance of AI in healthcare practices.

## Introduction

Artificial intelligence (AI) is the idea and practice of creating computer systems that can do tasks like speech recognition, decision-making, and pattern recognition that traditionally needed human intelligence. Natural language processing, machine learning (ML), deep learning (DL), and other technologies are all included under the broad term "artificial intelligence" [1].

The World Health Organization (WHO) provides a list of essential guidelines to guarantee the ethical and efficient application of AI. These include ensuring transparency and public understanding; promoting responsibility and accountability among stakeholders; ensuring inclusivity and equitable access; ensuring safety, accuracy, and public interest through stringent regulatory standards; ensuring human autonomy by upholding human control and guaranteeing privacy and informed consent; and fostering responsiveness and sustainability by minimizing environmental impacts and preparing the healthcare workforce for AI integration [2].

AI gadgets are mostly divided into two groups. ML approaches that analyze structured data, such as genetic, imaging, and EP data, fall under the first group. In medical applications, ML algorithms try to group characteristics of patients together or estimate the likelihood of a disease's course. Natural language processing (NLP) techniques fall under the second group. These techniques take information out of unstructured data, including clinical notes and medical journals, and use it to enhance and augment structured medical data. The goal of NLP processes is to convert texts into structured data that can be read by machines so that ML algorithms may analyze it [3].

AI has emerged as a transformative technology with the potential to revolutionize various sectors, including healthcare. AI encompasses a wide range of technologies, such as ML, NLP, and computer vision, which can be applied to healthcare practices to improve patient outcomes, optimize workflows, and reduce costs [4]. The adoption of AI in healthcare has gained momentum in recent years, with numerous applications being developed and implemented across various domains, such as diagnosis, treatment planning, drug discovery, and personalized medicine [5].

Healthcare professionals play a crucial role in the successful integration of AI into healthcare practices. Their knowledge, skills, and attitudes toward AI can significantly influence the adoption and effectiveness of these technologies [6]. Understanding the perception of healthcare professionals toward AI adoption is essential for identifying potential barriers, facilitators, and strategies for successful implementation [7].

Several studies have investigated the perception of AI among healthcare professionals. A survey conducted by Sit et al. [8] found that while healthcare professionals recognized the potential benefits of AI, they also expressed concerns about job security, lack of transparency, and the need for adequate training. Another study by Reddy et al. [9] highlighted the importance of involving healthcare professionals in the development and implementation of AI systems to ensure their trust and acceptance.

The adoption of AI in healthcare practices can offer numerous benefits. AI-powered diagnostic tools can assist healthcare professionals in making accurate and timely diagnoses, particularly in areas where human expertise is limited [10]. AI algorithms can analyze large volumes of patient data, including electronic health records, medical images, and genetic information, to identify patterns and predict outcomes [11]. This can enable early detection of diseases, personalized treatment plans, and improved patient monitoring.

However, the adoption of AI in healthcare also presents various challenges. One of the primary concerns is the potential for bias and discrimination in AI algorithms, which can perpetuate existing inequalities in healthcare [12]. Ensuring the transparency, interpretability, and accountability of AI systems is crucial to building trust among healthcare professionals and patients [13]. In addition, the integration of AI into healthcare practices requires significant investments in infrastructure, training, and change management.

Problems with AI implementation plague many healthcare organizations. Despite being widely used, including at the National Health Service (NHS), rule-based systems integrated into EHR systems are not as precise as more algorithmic systems based on ML. These rule-based clinical decision support systems are frequently unable to handle the explosion of data and knowledge based on genetic, proteomic, metabolic, and other "omic-based" approaches to care, and they are challenging to maintain as medical understanding advances [14].

To address these challenges and harness the full potential of AI in healthcare, it is essential to engage healthcare professionals in the process. By involving healthcare professionals in the design, development, and implementation of AI systems, we can ensure that these technologies align with their needs, values, and workflows. The present study aimed to assess the understanding of AI concepts, recognize the attitude of healthcare professionals towards the integration of AI into healthcare practices, and identify the barriers to the adoption of AI in healthcare.

## Materials and methods

Study setting, study population, and sampling method

A cross-sectional study was conducted at SRM Medical College Hospital and Research Centre, Chengalpattu district, Tamil Nadu, India. The study population included all healthcare professionals (doctors, nurses, and paramedical staff) of SRM Medical College Hospital who had experience of above six months and were available during the study period. Healthcare professionals who were not willing to participate in the study were excluded.

Simple random sampling was used to select the participants. All healthcare professionals who met specific eligibility criteria and showed their willingness to take part in the research were eligible for the study. The study was conducted over a period of three months from March to May 2024.

Sample size calculation

There were only a few studies examining healthcare professionals' perception of adoption of AI in healthcare practices and the proportion of healthcare professionals' perception of adoption in healthcare practices remained unknown. This study assumed that approximately 53.18% of the perception of the adoption of AI within their practice environment. To determine the necessary sample size for this study, the following formula for calculating sample size was employed: n = 4 PQ/D2 [15], where P = 53.18%, Q = 1-P, and D = 7%. The calculated sample size was 196.

Data collection procedure

The primary investigator collected data by conducting face-to-face interviews with healthcare professionals using a semi-structured standard questionnaire. The questionnaire is validated and reliable to use with IBM SPSS Statistics for Windows, Version 25.0 (released 2017, IBM Corp., Armonk, NY), showing a Cronbach’s alpha of 0.69. The questionnaire has four parts, namely, demographic data, questions related to knowledge in the adoption of AI, questions related to attitude in the adoption of AI, and questions related to barriers in the adoption of AI.

Statistical Analysis

All data were entered in Excel (Microsoft® Corp., Redmond, WA) and analyzed in SPSS Statistics version 25.0. Frequency analysis and percentage analysis were used to describe the data using descriptive statistics like sociodemographic data (age, sex, experience, and profession) for discrete variables. The scoring of knowledge, attitude, and barriers in the adoption of AI was calculated using mean and standard deviation. A mean value was calculated for each section, with scores below the mean considered low, scores equal to the mean considered moderate, and scores above the mean considered high. Scoring for knowledge ranges from 5 to 10, which is divided into three categories, i.e., low knowledge with a score less than 7, moderate knowledge with a score equal to 7, and high knowledge with a score more than 7. Scoring for attitude ranges from 3 to 6, which is divided into three categories, i.e., low attitude with a score of less than 5, moderate attitude with a score equal to 5, and high attitude with a score of more than 5. Scoring for barriers ranges from 9 to 18, which is divided into three categories, i.e., low barrier with a score of less than 12, moderate barrier with a score between 12 and 13, and high barrier with a score of more than 14. Factors associated with the perception of the adoption of AI were analyzed using a chi-square test, and the p-value was calculated. The level of statistical significance was set at 95% confidence interval. A probability value of less than 0.05 is considered significant.

Ethics approval

The SRM Institutional Ethics Committee granted its permission on March 20, 2024, and the ethical clearance number was obtained (SRMIEC-ST0224-911). After obtaining the ethical approval, the data collection was started in the SRM Medical College Hospital and Research Centre. Study participants provided informed consent.

## Results

Sociodemographic characteristics of the study participants

The study included a total of 200 healthcare professionals from SRM Medical College Hospital and Research Centre. Table 1 shows that the majority of the participants (75.5%,n = 151) were in the age group of 25 to 34 years, and the minority, i.e., 2.5% (n = 5) in the age group of 45 years and above. Among the study participants, 55% (n = 110) were female, and 45% (n = 90) were male. The distribution of work experience showed that 73% (n = 146) of the participants had one to five years of experience, 20.5% (n = 41) had six to 10 years of experience, and 6.5% (n = 13) had 11-20 years of experience. The study population consisted of 39.5% (n = 79) nurses, 37% (n = 74) paramedics, and 23.5% (47) doctors. The majority of the participants (96.5%, n = 193) were from Chennai, while 3.5% (n = 7) were from Chengalpattu.

**Table 1:** Distribution of the study participants according to the age, gender, work experience, designation, and address of healthcare professionals (N = 200)

	Classification	Frequency (n = 200)	Percentage (%)
Age	18 to 24 years	11	5.5
	25 to 34 years	151	75.5
	35 to 44 years	33	16.5
	45 years and above	5	2.5
Gender	Female	110	55
	Male	90	45
Work Experience	1-5 years	146	73
	6-10 years	41	20.5
	11-20 years	13	6.5
Profession	Doctors	47	23.5
	Nurses	79	39.5
	Paramedics	74	37
Address	Chennai	193	96.5
	Chengalpattu	7	3.5

Perception of the adoption of AI among healthcare professionals

Table 2 shows the frequency distribution of knowledge for the adoption of AI, revealing that 54% (n = 108) of the participants had moderate knowledge, 23.5% (n = 47) had low knowledge, and 22.5% (n = 45) had high knowledge. Regarding the attitude toward the adoption of AI, 48% (n = 96) of the participants had a low attitude, 34.5% (n = 69) had a moderate attitude, and 17.5% (n = 35) had a high attitude. The frequency distribution of barriers to the adoption of AI showed that 49% (n = 98) of the participants perceived low barriers, 44% (n = 88) perceived moderate barriers, and 7% (n = 14) perceived high barriers.

**Table 2:** Frequency distribution of knowledge, attitude, and barriers for the adoption of artificial intelligence (N = 200)

	Classification	Frequency	Percentage (%)
Knowledge	Low	47	23.5
	Moderate	108	54
	High	45	22.5
Attitude	Low	96	48
	Moderate	69	34.5
	High	35	17.5
Barrier	Low	98	49
	Moderate	88	44
	High	14	7

Knowledge, attitude, and barriers to AI in healthcare

Table 3 shows that a majority has knowledge about AI (95%, n = 190) and awareness of the AI technologies that have been currently implemented in your healthcare practices (82.5%, n = 165) and agree that uses of AI bring benefits for the patients (86.5%, n = 173). Regarding attitude, 64% (n = 128) have seen AI being used more in healthcare and 61% (n = 122) agree that physicians have adequate training to use AI on patients. Regarding barriers to the use of AI, 90.5% (n = 181) agree that securing funds for AI in healthcare faces hurdles; 86% (n = 172) participants believe that AI has the potential to reduce healthcare costs; 85% (n = 170) agree that through the use of AI, there will be fewer treatment errors in the future; and 81% (n = 162) accept that there is resistance among healthcare professionals toward the incorporation of AI.

**Table 3:** Knowledge, attitude, and barriers about artificial intelligence in healthcare

Questions	No		Yes	
	Frequency (n=200)	Percentage (%)	Frequency (n=200)	Percentage (%)
KNOWLEDGE				
Knowledge about Artificial Intelligence	10	5	190	95
Any work experience in AI field	157	78.5	43	21.5
Awareness with the AI technologies that has been currently implemented in your healthcare practices	35	17.5	165	82.5
Uses of AI brings benefits for the patients	27	13.5	173	86.5
Received formal training on AI applications in healthcare	163	81.5	37	18.5
ATTITUDE				
Have seen AI being used more in healthcare	72	36	128	64
Practiced AI tools into your daily healthcare routines	162	81	38	19
Physicians have adequate training to use AI on patients	78	39	122	61
BARRIERS				
Securing funds for AI in healthcare faces hurdle	19	9.5	181	90.5
Lack of training is a barrier for adopting AI in healthcare	77	38.5	123	61.5
Perceived risks associated with patient data security in AI enabled healthcare	105	52.5	95	47.5
Have job displacement concern due to AI in healthcare	129	64.5	71	35.5
The resistance among healthcare professionals towards the incorporation of AI	38	19	162	81
AI is changing the demands of the medical profession	139	69.5	61	30.5
Through the use of AI, there will be less treatment errors in the future	30	15	170	85
Enough workforce for AI in healthcare	88	44	112	56
Believe that AI has the potential to reduce healthcare costs	28	14	172	86

Association between knowledge and other factors influencing AI

The association between knowledge and attitude was analyzed using the Chi-square test. In Table 4, the results showed a statistically significant association between knowledge and attitude (X² = 18.052, df = 4, p = 0.001). Healthcare professionals with moderate knowledge levels had low attitudes toward the adoption of AI.

**Table 4:** Association between knowledge and attitude toward the adoption of artificial intelligence in healthcare professionals

Knowledge	Attitude			X²	P Value
	Low	Moderate	High		
Low	32 (68.1)	10 (21.3)	5 (10.6)	18.052(4)	0.001
Moderate	52 (48.1)	40 (37.0)	16 (14.8)		
High	12 (26.7)	19 (42.2)	14 (31.1)		

Table 5 reveals the association between knowledge and barriers as analyzed using the Chi-square test. The results indicated a statistically significant association between knowledge and barriers (X² = 31.235, df = 4, p = 0.00). Healthcare professionals with higher knowledge levels perceived lower barriers to the adoption of AI.

**Table 5:** Association between knowledge and barriers toward the adoption of artificial intelligence in healthcare professionals

Knowledge	Barriers			X²	P-value
	Low	Moderate	High		
Low	36 (76.6)	10 (21.3)	1 (2.1)	31.235	0.000
Moderate	38 (35.2)	64 (59.3)	6 (5.6)		
High	24 (53.3)	14 (31.1)	7 (15.6)		

Table 6 shows the association between attitude and barriers was analyzed using the Chi-square test. The results showed no statistically significant association between attitude and barriers (X² = 8.866, df = 4, p = 0.065).

**Table 6:** Association between attitude and barriers toward the adoption of artificial intelligence in healthcare professionals

Attitude	Barriers			X²	P-value
	Low	Moderate	High		
Low	57 (59.4)	35 (36.5)	4 (4.2)	8.866 (4)	0.065
Moderate	28 (40.6)	35 (50.7)	6 (8.7)		
High	13 (37.1)	18 (51.4)	4 (11.4)		

## Discussion

The findings of this study provide valuable insights into the perception of AI adoption among healthcare professionals in a tertiary care hospital setting.

The study found that 54% of the participants had moderate knowledge about the adoption of AI in healthcare, while 23.5% had low knowledge and 22.5% had high knowledge. These findings are comparable to a study by Scheetz et al. [6], which reported that 53% of the clinicians had moderate knowledge about AI applications in their respective fields.

The majority of the study participants (75.5%) were in the age group of 25 to 34 years, which is consistent with the findings of a similar study conducted by Sit et al. [8], where 68% of the participants were in the same age group. This suggests that younger healthcare professionals are more likely to participate in studies related to AI adoption in healthcare.

Regarding the attitude toward AI adoption, 48% of the participants in the present study had a low attitude, 34.5% had a moderate attitude, and 17.5% had a high attitude. These findings are in contrast with a study by Sit et al. [8], which found that 63% of medical students had a positive attitude toward AI in radiology. By contrast, the study by Queralt Miro Catalina et al. showed that 85.7% of participants understood the concept of AI, although 65.8% had not received any AI training and 91.4% expressed a desire for training. In addition, the mean score for the professional impact of AI was 3.62 out of 5, with those having prior knowledge and interest in AI rating it higher. Preparedness for AI had a mean score of 2.76 out of 5, with higher scores among nursing professionals and those unsure if they used AI [16]. The difference in attitudes may be due to the varying levels of exposure and understanding of AI among different healthcare professionals and students.

The present study identified that 49% of the participants perceived low barriers, 44% perceived moderate barriers, and 7% perceived high barriers to the adoption of AI in healthcare. A study by Ahmed MI et al. [17] reveals that despite AI technologies being used in healthcare for some decades and all the theoretical potential of AI, the uptake in healthcare has been uneven and slower than anticipated and there remain a number of barriers, both overt and covert, which have limited its incorporation.

The study found a statistically significant association between knowledge and attitude toward AI adoption (X² = 18.052, df = 4, p = 0.001). Healthcare professionals with higher knowledge levels had a more positive attitude toward AI adoption. This is similar to a study by Ahmed Z et al. [18]. The results show a significant association, with a higher level of knowledge correlating with more positive attitudes. The study's statistical findings (X² = 16.45, df = 4, p < 0.01) are similar to those you described. This finding is consistent with a study by Tran et al. [19], which reported a significant positive correlation between AI knowledge and attitude (r = 0.39, p < 0.001). This highlights the importance of providing adequate education and training to healthcare professionals to foster a positive attitude toward AI adoption.

Furthermore, the present study found a statistically significant association between knowledge and perceived barriers to AI adoption (X² = 31.235, df = 4, p = 0.00). Healthcare professionals with higher knowledge levels perceived lower barriers to AI adoption. This finding is supported by a study by Shaw et al. [20], which found that healthcare professionals with higher AI knowledge reported fewer barriers to its implementation (OR = 0.68, 95% CI: 0.52-0.89, p < 0.01). This emphasizes the need for addressing knowledge gaps and providing necessary training to overcome the perceived barriers to AI adoption in healthcare.

However, the study did not find a statistically significant association between attitude and perceived barriers to AI adoption (X² = 8.866, df = 4, p = 0.065). This finding is in contrast with a study by Golinelli et al. [21], which reported a significant negative association between attitude and perceived barriers (β = -0.24, p < 0.01). The discrepancy in the findings may be due to differences in the study populations, healthcare settings, and the complex interplay of factors influencing attitudes and perceived barriers.

In our study, the population consisted of the majority of nurses (39.5%, n = 79), followed by paramedics (37%, n = 74) and doctors (23.5%, 47), which is similar to a study done by Shinners L et al., which showed that participants represented "non-clinical roles" (n = 24, 9.5%) and "clinical roles," including nursing/midwifery, the majority (n = 141, 56%), medicine (n = 34, 13.5%), allied health professionals (n = 26, 10.3%), and other healthcare professionals (n = 27, 10.7%) [22].

The present study has several strengths, including a representative sample of healthcare professionals from a tertiary care hospital and the use of a validated questionnaire (SPSS software version 25 shows a Cronbach’s alpha of 0.69) to assess the perception of AI adoption. Futuristic ideas about AI and their barrier approaches are discussed in our study, which can be worked upon to improve healthcare efficacy. However, the study also has some limitations. First, the study was conducted only for a period of three months (March 2024-May 2024). This will impact the depth of analysis and exploration of all relevant factors associated with the quality of care, and the findings may not be generalizable to other healthcare settings because they might have other perceptions of adopting AI in their daily healthcare practices. Conducting multicenter studies could provide a broader understanding of how healthcare professionals are adopting AI in their daily practices.

## Conclusions

This study provides valuable insights into the perception of AI adoption among healthcare professionals in a tertiary care hospital. AI has the potential to improve healthcare and organizational efficiency, but achieving this full potential will require skillfully navigating technical obstacles, regulatory difficulties, and ethical issues. The findings highlight the need for adequate education and training to improve knowledge, foster positive attitudes, and address the perceived barriers to AI adoption in healthcare.

Future research should focus on developing and evaluating targeted interventions to enhance the understanding and acceptance of AI among healthcare professionals, ultimately leading to the successful integration of AI in healthcare practices. Furthermore, studies should explore the impact of AI adoption on patient outcomes, healthcare efficiency, and the evolving roles of healthcare professionals in the era of AI.
